# Hyperspectral Imaging Reveals Differential Carotenoid and Chlorophyll Temporal Dynamics and Spatial Patterns in Scots Pine Under Water Stress

**DOI:** 10.1111/pce.15225

**Published:** 2024-10-27

**Authors:** Iiro Miettinen, Chao Zhang, Luis Alonso, Beatriz Fernández‐Marín, José I. García‐Plazaola, Steffen Grebe, Albert Porcar‐Castell, Jon Atherton

**Affiliations:** ^1^ Optics of Photosynthesis Laboratory, Department of Forest Sciences, Institute for Atmospheric and Earth System Research (INAR) Faculty of Agriculture and Forestry, University of Helsinki Helsinki Uusimaa Finland; ^2^ Fundanción CEAM Paterna Valencia Spain; ^3^ Department of Plant Biology and Ecology University of the Basque Country (UPV/EHU) Leioa Basque Country Spain

**Keywords:** drought, hyperspectral imaging, photochemical reflectance index, red edge, remote sensing, Scots pine, water stress, xanthophyll cycle

## Abstract

Drought‐related die‐off events have been observed throughout Europe in Scots pine (*Pinus sylvestris* L.). Such events are exacerbated by carbon starvation that is, an imbalance of photosynthetic productivity and resource usage. Recent evidence suggests that optically measurable photosynthetic pigments such as chlorophylls and carotenoids respond to water stress (WS). However, there is a lack of measurements using imaging spectroscopy, and the mechanisms linking xanthophyll‐related changes in reflectance captured by the photochemical reflectance index (PRI) and chlorophyll changes in red edge position (REP) to WS are not understood. To probe this, we conducted a greenhouse experiment where 3‐year‐old *Pinus sylvestris* saplings were subjected to water limitation and followed using hyperspectral imaging (HSI) spectroscopy, water status and photosynthetic measurements. Carotenoids (e.g., xanthophyll cycle) and chlorophylls responded to WS, which was observed using the HSI‐derived indices PRI and REP respectively. The spatial‐temporal response in these two pigment‐reflectance groupings differed. The spatial distribution of PRI represented the light intensity around the time of the measurement, whereas REP reflected the daily averaged light intensity over the experimental course. A further difference was noted upon rewatering, where the carotenoid‐related PRI partially recovered but the chlorophyll‐related REP did not.

## Summary statement

Hyperspectral imaging spectroscopy was used to detect water stress‐induced changes in Scots pine reflectance. Differential carotenoid and chlorophyll‐related responses were found, probably related to the experimental light environment.

**Abbreviations**
DEPS: xanthophyll cycle de‐epoxidation state
HSI: hyperspectral imaging
NPQ: non‐photochemical quenching
PQ: photochemical quenching
PRI: photochemical reflectance index
qLr: reversible component of photochemical quenching
qLs: sustained component of photochemical quenching
qLT: photochemical quenching according to the lake model
REP: red edge position
WS: water stress
Φ*NPQ*_*R*_
: quantum yield of reversible non‐photochemical quenching
Φ*NPQ*_*S*_
: quantum yield of sustained non‐photochemical quenching
Φ*PSII*
: quantum yield of PSII photochemistry in light
Φ*PSII*_*MAX*_
: maximum quantum yield of PSII photochemistry
*Ψ_W_*
: leaf water potential

## 1 Introduction

Widely distributed across much of Europe and Asia, Scots pine (*Pinus sylvestris* L.) is especially prevalent in northerly latitudes (Brichta et al. 2023). Several studies have reported drought‐related decline of *P. sylvestris* across southern and central Europe (Vertui and Tagliaferro 1998; Rigling and Cherubini 1999; Bigler et al. 2006; Bréda et al. 2006; Wermelinger et al. 2008; Hereş, Martínez‐Vilalta, and Claramunt López 2012). In northern Europe, where *P. sylvestris* is more susceptible to winter drought (Aldea et al. 2024), recent reports have also pointed to drought‐related mortality events (Mäkinen, Nöjd, and Helama 2022; Junttila et al. 2024), which are probably exacerbated by insect or fungal interactions (Nuorteva et al. 2023; Ylioja et al. 2023).

The extent and magnitude of water stress (WS) related decline depends on site conditions and the history of the individual trees, suggesting that small‐scale monitoring is required for successful management and mitigation (Bose et al. 2020). Optical remote sensing can potentially meet this need, and modern platforms, such as drones or CubeSats, can resolve tree targets at the spatial scales required for individual‐based monitoring (D'Odorico et al. 2020, 2021; Konings et al. 2021). However, the link between WS and optical remote sensing is far from straightforward and requires careful consideration of the downregulation of photosynthesis before WS can be detected previsually from remote sensing platforms.

WS induced tree mortality can be caused by hydraulic failure, which occurs due to the cavitation of water transporting vessels (McDowell et al. 2008; Galiano, Martínez‐Vilalta, and Lloret 2011). To reduce the risk of cavitation, *P. sylvestris* readily close their stomata to lessen water loss from transpiration in response to declining soil moisture content (Irvine et al. 1998; Dulamsuren et al. 2009). This stomatal closure reduces the intake of CO_2_, which hinders photosynthetic activity. Moreover, WS impacts photosynthesis by non‐stomatal limitations by decreasing mesophyll conductance and biochemical capacity (Flexas et al. 2014; Urban, Aarrouf, and Bidel 2017). The water saving strategy of *P. sylvestris* therefore reduces photosynthesis in favour of losing shoot water potential, probably to reduce the chances of xylem embolism (Dulamsuren et al. 2009). The subsequent reduction of photosynthetic capacity predisposes *P. sylvestris* to carbon starvation, which occurs when the sink usage of assimilates outweigh the downregulated photosynthetic production—if left unchecked this could eventually result in death (McDowell et al. 2008; Galiano, Martínez‐Vilalta, and Lloret 2011; Poyatos et al. 2013; Aguadé et al. 2015).

The isohydric WS response strategy of *P. sylvestris* reduces the consumption of photosynthetic energy by the light‐independent reactions (Luoma 1997; Dulamsuren et al. 2009) resulting in an excess of absorbed energy or excitation pressure which can lead to the formation of reactive oxygen species, which cause oxidative damage (Cruz de Carvalho, 2008). To evade damage, this excitation pressure can be dissipated as heat by non‐photochemical quenching (NPQ). Components of NPQ can be classified based on their temporal dynamics as reversible and sustained (Demmig‐Adams and Adams 2006; Porcar‐Castell 2011). Reversible NPQ refers to processes that are readily reversed in the dark. Reversible NPQ is associated with the building of a trans‐thylakoid proton gradient, protonation of PSII proteins, presence of the Psbs protein, and the de‐epoxidation of the carotenoid violaxanthin to zeaxanthin via antheraxanthin, which constitutes the xanthophyll cycle. In contrast, sustained NPQ does not revert in the dark overnight and has been associated with aggregation of light‐harvesting proteins, photoinhibition of PSII reaction centres, and sustained build‐up of zeaxanthin (Fernández‐Marín et al. 2021).

Changes in the foliar concentration of pigments related to NPQ can be detected using imaging spectroscopy (Rascher et al. 2007), which is both nondestructive and easily scalable compared to typical laboratory and field sampling protocols. Notably, the reversible de‐epoxidation state of the xanthophyll cycle (DEPS) can be estimated via an optical proxy—the photochemical reflectance index (PRI) (Gamon, Peñuelas, and Field 1990, 1992). The utility of PRI as an indicator of stress induced by water limitation has been demonstrated on various scales using mainly broadleaf trees (Suárez et al. 2008; Ripullone et al. 2011; Zhang et al. 2017). In a recent study D'odorico et al. (2021) reported moderate correlations between DEPS and PRI, and the more slowly changing carotenoid/chlorophyll ratio and PRI, from drone‐based multispectral imaging spectroscopy in *P. sylvestris* across irrigated and water‐limited treatments.

In addition to carotenoid pigments, foliar chlorophyll content of *P. sylvestris* has been reported to decrease under water deficit conditions (Zlobin, Kartashov, et al. 2019). Furthermore, several studies have related chlorophyll‐specific optical vegetation indices to forest drought in the field (Grulke et al. 2020; Moreno‐Fernández et al. 2021; Raddi et al. 2022). Presumably, these studies are detecting chlorosis and degradation or an adaptive response of chlorophyll that is distinct from the reversible NPQ mechanisms discussed above. Chlorophyll‐related optical indices typically rely on the red‐edge feature, which is the rapid increase in leaf reflectance between visible and near‐infrared ranges caused by chlorophyll absorbance (Filella and Peñuelas 1994). The inflection point of the red‐edge feature, that is, the red‐edge position (REP), has been successfully used as a proxy for chlorophyll content in multiple species, conditions and scales (Filella and Peñuelas 1994; Cho and Skidmore 2006), including in WS monitoring studies (Zhang et al. 2017; Raddi et al. 2022). Notably, most of these studies have had a focus on broadleaf species, and it is hence unclear if WS induced variability, or degradation, in chlorophyll is detectable using optical methods in *P. sylvestris*.

Factors such as leaf inclination angles (Xu et al. 2021), soil scattering, solar‐view geometry, shadow masking and atmospheric conditions influence the resulting signal in remote sensing studies. These factors potentially conceal fine changes related to pigments. A greenhouse study is therefore required to probe downregulation‐related processes in more detail for example, intratree variation in optical properties. Furthermore, to our knowledge no other study has followed the photosynthetic pigments of *P. sylvestris* on resumption of watering over shorter timescales, although a legacy effect in mature *P. sylvestris* that were previously subjected to water deficits has been reported (D'Odorico et al. 2021).

Our aim was to use hyperspectral imaging spectroscopy (HSI) to follow the development and subsequent relaxation of WS in *P. sylvestris* over a few weeks. We hypothesized that we would observe WS‐related photosynthetic downregulation in the xanthophyll cycle‐sensitive PRI but also in the chlorophyll‐specific REP. We also hypothesized that changes in the PRI would relate to the reversible xanthophyll cycle and in turn to reversible NPQ. Our final hypothesis was that we would find spatial changes within trees in PRI and REP that developed in relation to the local light environment. To test these hypotheses, we conducted a greenhouse‐based water deficit experiment where we combined HSI with pigment sampling and photosynthetic measurements on young *P. sylvestris* trees.

## 2 Materials and Methods

### 2.1 Experimental Design

The experiment was conducted in a greenhouse setting at the University of Helsinki Viikki campus in Helsinki, Finland. The plant material consisted of eight 3‐year‐old *P. sylvestris* L. nursery saplings that were 54–63 cm high after the current growth season elongation (18–23 cm) had finished. The plants were grown in individual pots in peat potting substrate. Before the start of the experiment, the plants had been adapted to greenhouse conditions since mid‐May.

**Figure 1:**
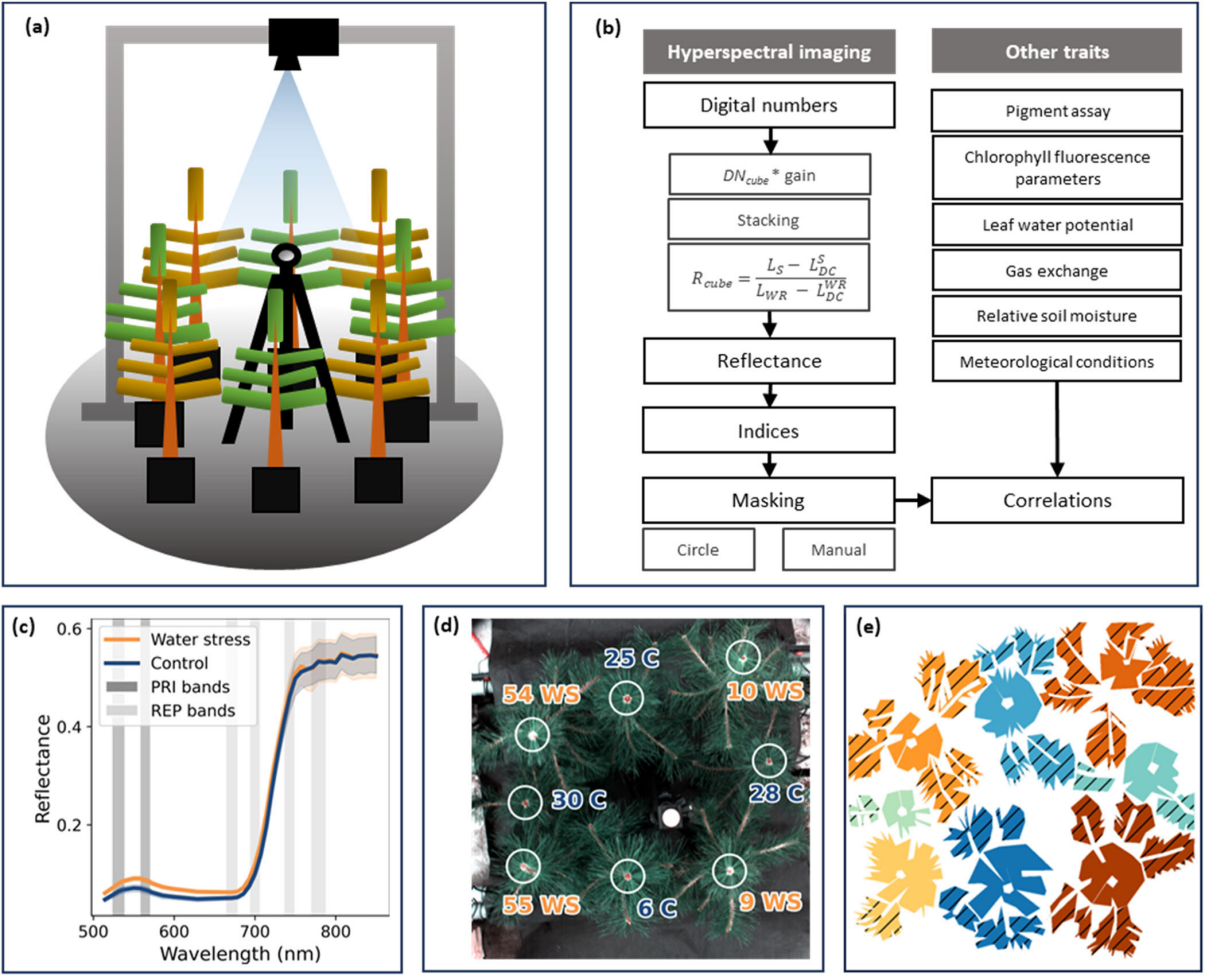
(a) Illustration of the imaging set‐up showing sample trees, white reference panel on a tripod, and the HSI camera pointing downwards. (b) Flowchart of data acquisition, processing and analysis. (c) Average spectra of WS and control plants during the height of WS as detected by HSI with bands used to calculate the photochemical index (PRI) and red edge position (REP). (d) RGB image of saplings constructed from hyperspectral bands with plant ID and treatment labels—‘WS' for water stress and ‘C' for control, and circle masks over primary shoots. (e) Manually drawn masks with plants separated by colour and shoot location by pattern.

The experiment took place in August and September 2022, by which time the current year needles had reached maturity. Half of the saplings were randomly assigned to undergo water limitation treatment, during which irrigation was withheld completely for 17 days (15–31 August 2022). The remaining half functioned as controls and were watered every morning by hand on measurement days with approximately the same dose. Position and group (control or WS) were assigned randomly to plants (Figure 1d). Physiological pre‐WS measurements were performed on the 9th, 12th, and 15th August 2022, and pre‐WS imaging was done on the 15th August. After irrigation was resumed, recovery monitoring was carried out for 7 days.

Gas exchange and light‐adapted fluorescence was measured on 3 days: pre‐WS (9 August) between 12:20 and 16:00, height of WS (31 August) between 12:20 and 17:00, and end of recovery monitoring (8 September) between 11:20 and 17:20. Pigments were sampled on 2 days: peak of WS (31 August), and end of recovery (8 September) at 12:00 to 12:10 on both days. Soil moisture (SM‐%), leaf water potential (*Ψ_W_*), and dark‐adapted chlorophyll fluorescence were measured along with hyperspectral imaging on 17 days. Measurements of SM‐%, *Ψ_W_*, and dark‐adapted chlorophyll fluorescence took place in the morning between 8:40 and 11:50, while hyperspectral imaging was carried out around midday between 10:50 and 13:10. Hyperspectral imaging was timed around midday to minimize shading from greenhouse infrastructure.

### 2.2 Environmental Measurements

Photosynthetically active radiation (PAR, µmol photons m^−2^ s^−1^) in the greenhouse was recorded manually during HSI measurements using a Li‐250A Light Metre with a quantum sensor (Li‐Cor, Lincoln, NE, USA) mounted on the experiment set‐up to the height of the HSI camera. Note that the light conditions in the greenhouse were diffused with sheer curtains for temperature control, which reduced PAR inside the greenhouse relative to outdoor values. Temperature in the greenhouse was monitored every 5 min. Generally, the conditions in the greenhouse varied as a function of the outside weather, as the roof window vents were open. Relative soil moisture was monitored using an HH2 Soil Moisture Meter with a ML3 ThetaProbe sensor (Delta‐T Devices Ltd., Cambridge, UK) at the depth of 10 cm in three locations per pot.

### 2.3 Chlorophyll Fluorescence Parameters

Minimal (*F*_0_) and maximal fluorescence (*F*_*M*_) of dark acclimated needles was measured using a pulse‐amplitude‐modulation (PAM) chlorophyll fluorometer PAM‐2500 (Heinz Walz GmbH, Effeltrich, Germany). PAM measurements were performed on healthy top shoot needle mats that were dark acclimated for at least 40 min. Within 1 day, PAM measurements were carried out in a 15‐min window between approximately 9:20 and 11:50. Three repeat measurements per sample plant were taken.

The maximum quantum yield of PSII photochemistry (Φ*PSII*_*MAX*_) was calculated after Genty, Briantais and Baker (1989). Additionally, the quantum yields of sustained, reversible and total non‐photochemical quenching (Φ*NPQ*_*s*_, Φ*NPQ*_*R*_ and Φ*NPQ*) were calculated with the corresponding rate constants (*K*_*NPQs*_, *K*_*NPQr*_ and *K*_*NPQ*_) after Porcar‐Castell (2011). The parameter for photochemical quenching according to the lake model (qLT) (Kramer et al. 2004) was calculated alongside sustained and reversible PQ parameters qLr and qLs after Porcar‐Castell (2011). Equations for all chlorophyll fluorescence parameters are available in the appendices (Equations (A1), (A2), (A3), (A4), (A5), (A6), (A7), (A8), (A9), (A10), (A11)).

Required for some chlorophyll fluorescence parameters, the reference maximal fluorescence value (*F*_*MR*_) was determined by plant‐wise linear regression of *F*_*M*_ and Φ*PSII*_*MAX*_ and by finding *F*_*M*_ for a maximal reference Φ*PSII* set at 0.85 to represent a season‐maximum. Reference minimal fluorescence (*F*_0*R*_) was determined based on the maximal reference Φ*PSII* and *F*_*MR*_ (Equation A12).

### 2.4 Gas Exchange and Additional Fluorescence Parameters

Gas exchange measurements were conducted using the portable gas exchange and fluorescence system GFS‐3000 (Heinz Walz GmbH, Effeltrich, Germany) on three to four pairs of needles, which were organized in an 8 cm^2^ measuring cuvette. To prevent gas leaks between the needle and cuvette gasket, mouldable wax earplugs were used to seal the needles into place. The measurement environment inside the cuvette was set as follows: flow rate was 650 µmol s^−1^, CO_2_ concentration was 420 ppm, relative humidity was 60%, cuvette temperature was 25°C, and quantum flux density was 1000 µmol m^−2^ s^−1^. The steady state measurement was recorded every 2 s for 2 min and used for data analysis. At the end of the measurement, maximal fluorescence under a saturating pulse *F'*_*M*_ and steady‐state fluorescence *F'*_*S*_ were measured under a white LED measuring light at light intensity of 1000 µmol m^−2^ s^−1^.

After each measurement, a photo was taken above the closed cuvette to obtain leaf area for area‐based correction. The photo was first rectified by using the image rectification tool (https://smallpond.ca/jim/scale/rectify.html), then the dual‐sided leaf area was calculated using Fiji‐ImageJ software (Schneider, Rasband, and Eliceiri 2012). CO_2_ assimilation rate (*A*, µmol m^−2^ s^−1^) and transpiration rate (*E*, mmol m^−2^ s^−1^) were estimated after Von Caemmerer and Farquhar (1981).

### 2.5 Leaf Water Potential

Leaf water potential (*Ψ_W_*, MPa) was measured using a model 1505D‐EXP pressure chamber (PMS Instrument Company, Albany, OR, USA). The method described in Scholander et al. (1965) was followed with adjustments made to accommodate pine needles. For each sample two to four replicates were measured. Each replicate consisted of a healthy current‐year needle pair collected from lateral shoots. Needles were collected between 9:00 and 10:00 am and measured immediately.

### 2.6 Pigment Analysis

Needles were collected for pigment analysis during height of WS (31 August) and at the end of the recovery period (8 September) at 12:00 to 12:10 on both days concurrently with HSI. The samples were collected from healthy top shoot needles and the apexes of the needles were excluded. Samples were placed in liquid nitrogen immediately, after which they were placed in −80°C for long‐term storage.

Pigments were analysed using a reverse‐phase Waters (Milford, MA, USA) high‐performance liquid chromatography (HPLC) system following the method of García‐Plazaola and Becerril (1999) with the modifications described in García‐Plazaola and Becerril (2001) using 15 μL of sample extract. A photodiode array (PDA) detector (Waters model 996) was used to identify and quantify photosynthetic pigments (violaxanthin, antheraxanthin, zeaxanthin, lutein, lutein epoxidate, chlorophyll a and b, and α‐ and β‐carotene) in the range 250–700 nm. Peaks were detected and integrated at 445 nm. Identification and quantification were conducted by comparison with pure commercial standards.

Concentrations of pigments were determined based on needle area (µg cm^−2^). Area calculation assumed that a needle was a hemicylinder. The length of sample was set at 5 cm, which corresponded to 70−100 mg in fresh weight, and needle width was measured based on a photograph with ImageJ software (Schneider, Rasband, and Eliceiri 2012). Pigment ratios and the de‐epoxidation state of the xanthophyll pigments (DEPS) were calculated using molar concentrations (mol per sample). DEPS was calculated after Demmig‐Adams and Adams (1996) as:

(1) $$\text{DEPS}=\frac{A+Z}{V+A+Z}.$$

### 2.7 Hyperspectral Imaging

Hyperspectral imaging was conducted using a Senop HSC‐2 Hyperspectral camera (Senop Ltd., Kangasala, Finland), which has two detectors (VIS and NIR), which are based on Fabry‐Perot interferometer technology and function within the combined wavelength range of 510–900 nm. Hyperspectral images were taken nadir to sapling canopy, so that the distance from the camera lens to top shoot was 97–108 cm at an off‐nadir angle of 8.4–19.7°. The white reference was placed on a tripod at median plant height at the centre of the scene. The white reference used was a Spectralon Diffuse Reflectance Standard (Labsphere, Sutton, NH, USA) with 99% reflectance over a 250–2500 nm wavelength range. HSI was done under ambient greenhouse light conditions, which were diffused by a curtain. Photosynthetically active photon flux during HSI varied day‐to‐day between 80 and 330 μmol m^−2^ s^−1^. The imaging set‐up is illustrated in Figure 1a, and the HSI capturing and processing protocol is summarized in Figure 1b.

The imaging protocol consisted of eight data‐cubes with four different integration times. Half of the cubes contained images of the samples, and the rest were dark current measurements with corresponding integration times. The four integration times included in the imaging protocol were manually optimized for vegetation and the white reference separately for the two detectors in the Senop HSC‐2 camera. Furthermore, integration times were readjusted between measurements in response to changing light conditions. One HSI cube consisted of 48 evenly distributed bands centred between 514 and 850 nm and PRI bands centred at 531 and 570 nm with the narrowest available bandwidth selected. The bands' full width at half maximum was 13–19 nm. The imaging protocol was devised and executed using the Senop HSI‐2 software.

In postprocessing, saturated pixels were removed first. Subsequently, the data cubes were normalized by applying the band‐wise gain factor provided in the image header, which converted the images to radiance units (mW m^−2^ nm^−1^ sr^−1^). The gain‐corrected images were stacked and averaged together. The reflectance cube was attained by dividing the radiance sample cube ($L_{S}$) by the white reference spectrum ($L_{\mathrm{WR}}$) after subtracting the corresponding dark currents ($L_{\mathrm{DC}}^{S}$ and $L_{\mathrm{DC}}^{\mathrm{WR}}$) as follows:

(2) $$R_{\mathrm{cube}}=\frac{L_{S}-L_{\mathrm{DC}}^{S}}{L_{\mathrm{WR}}-L_{\mathrm{DC}}^{\mathrm{WR}}},$$

in which all L radiance cubes were in mW m^−2^ nm^−1^ sr^−1^.

Due to measuring under diffused sunlight, we did not correct the white panel with laboratory measured and manufacturer‐supplied reflectance coefficients. $L_{\mathrm{DC}}^{S}$ was calculated for all sample cubes with the corresponding integration time by averaging the dark current cube over the spectral dimension, since all bands are acquired by the same sensor elements. $L_{\mathrm{DC}}^{\mathrm{WR}}$ was calculated in the same manner, but with integration time corresponding to the WR images. $L_{\mathrm{WR}}$ was estimated by subsetting white reference pixels in the cube with the shortest integration time available to avoid saturated pixels, and then averaging them band by band.

Optical vegetation indices were calculated based on the processed reflectance cubes. The photochemical reflectance index (PRI) was calculated after Gamon, Peñuelas and Field (1992) as:

(3) $$\mathrm{PRI}=\frac{R_{531}-R_{570}}{R_{531}+R_{570}},$$

in which *R* was the reflectance of a band centred at a specified wavelength.

The red edge position (REP) was estimated by linear four‐point interpolation (Cho and Skidmore 2006) using the nearest band available in the cube:

(4) $$\mathrm{REP}(\mathrm{nm})=700+40\;\frac{R_{i}-R_{700}}{R_{740}-R_{700}},$$

in which and *R*_i_ was:

(5) $$R_{i}=\frac{R_{670}+R_{780}}{2}.$$

The nearest REP bands available in the imaging sequence were centred at 700, 742.9, 671.4 and 778.6 nm.

### 2.8 Statistical Analyses

Pearson's correlation coefficient (*r*) was calculated between all variables. Wilcoxon rank‐sum tests were used to test the differences in distributions between water stress and control within days. Effect size was calculated as Cohen's standardized mean difference (Cohen's *d*) for comparison of optical indices between shoots within one plant on specific days to test for intra‐tree heterogeneity. Sets used to calculate Cohen's *d* were normally distributed, and their variance was homogeneous (*σ* ^2^_A_/*σ* ^2^_B_ > 14) enough to not violate the assumptions of Cohen's *d* (Li 2016). Analysis of optical indices was based on average value over a circle mask (see Figure 1d) that encompassed the illuminated top of the primary shoot, with the exception of intra‐tree heterogeneity, which was investigated based on manually drawn masks (Figure 1e). Statistical analysis was conducted using Python (v3.11).

## 3 Results

### 3.1 Water Stress Development and Water Status Indicators

**Figure A1:**
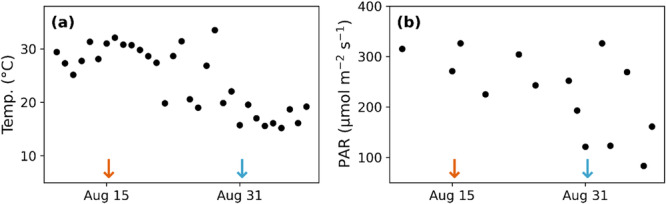
Meteorological data from the experiment's duration, including (a) midday temperature in the greenhouse and (b) midday PAR in the greenhouse at the time of HSI.

The greenhouse temperature ranged between 6°C and 35°C during the experiment (9 August to 8 September). The pre‐WS and the WS treatment period coincided with a heatwave, during which temperature in the greenhouse regularly climbed above 30°C. Greenhouse midday temperature at the end of WS treatment and during recovery remained below 20°C. The greenhouse PAR ranged from 80 to 330 μmol m^−2^ s^−1^ and averaged at 230 μmol m^−2^ s^−1^. In comparison, the outside PAR ranged from 420 to 2160 μmol m^−2^ s^−1^ often being an order of magnitude higher than in the greenhouse. Midday temperature and PAR in the greenhouse are shown in Figure A1a,b.

**Figure 2:**
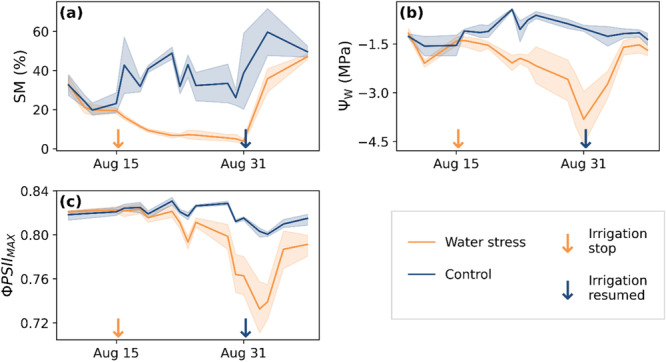
Timeseries of (a) relative soil moisture (SM‐%), (b) leaf water potential (*Ψ_W_*) in MPa, and (c) maximum quantum yield of photosystem II photochemistry Φ*PSII*_*MAX*_. Solid lines denote mean, and the fill area confidence interval (95%). The orange arrow points to the stop of irrigation (August 15) and the blue arrow to the restart of irrigation (August 31). [Color figure can be viewed at wileyonlinelibrary.com]

Relative soil moisture (SM‐%) of control and WS plants was between 17% and 31% at the start of the experiment. By the end of the WS period, WS plant SM‐% had decreased to 2.4%−5.8% (Figure 2a). The difference in leaf water potential (*Ψ_W_*) between control and WS plants was significant from 16 August till 3 September according to a Wilcoxon rank‐sum test with a 95% confidence interval. Difference in the distribution of *Ψ_W_* was nonsignificant during pre‐WS and from 5 September till the end of the recovery monitoring period (Figure 2b). SM‐% of two WS plants increased ca. 1%−3% on 24 August due to a leak in the greenhouse roof. Following this, variance of WS plant SM‐% and *Ψ_W_* increased in the middle of the WS treatment period.

### 3.2 Chlorophyll Fluorescence

The maximum quantum yield of PSII photochemistry (Φ*PSII*_*MAX*_) of control plants ranged between 0.80 and 0.84 during the experiment. In comparison, WS plant Φ*PSII*_*MAX*_ ranged between 0.70 and 0.83 during WS, and hence decreased modestly but significantly (Wilcoxon rank‐sum *p* > 0.05). After irrigation was restarted, WS Φ*PSII*_*MAX*_ increased rapidly, but did not reach a level equivalent to the control plants (Figure 2c). Control plant *ΦPSII*_*MAX*_ remained stable throughout the experiment until it experienced a dip that coincided with the recovery monitoring period and a drop in temperature. The variance of control plant Φ*PSII*_*MAX*_ (*σ* ^2^ < 1e^−5^) was smaller in comparison to WS plants (*σ* ^2^ = 0.02) during the height of WS, which demonstrated less variability between individuals under favourable conditions.

**Figure A2:**
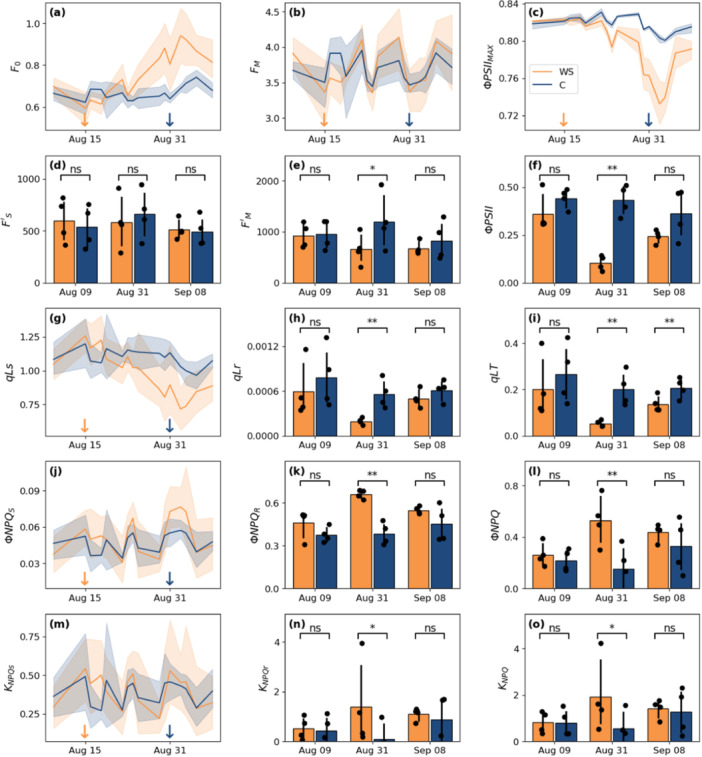
Timeseries of (a) minimal fluorescence of a dark‐adapted sample (*F*_0_), (b) maximal fluorescence of a dark‐adapted sample (*F_M_*), (c) quantum yield of maximal of PSII photochemistry (Φ*PSII*_MAX_), (d) instantaneous fluorescence (*F'_S_*), (e) maximal fluorescence of an in‐light sample (*F’_M_*), (f) quantum yield of PSII photochemistry (Φ*PSII*), (g) sustained photochemical quenching parameter qLs, (h) reversible photochemical quenching parameter qLr, (i) total photochemical quenching parameter qLT, (j) quantum yield of sustained non‐photochemical quenching (Φ*NPQ*_*S*_), (k) quantum yield of reversible non‐photochemical quenching (Φ*NPQ*_*R*_), (l) quantum yield of non‐photochemical quenching (Φ*NPQ)*, (m) rate constant of sustained non‐photochemical quenching (*K*_*NPQs*_), (n) rate constant of reversible non‐photochemical quenching (*K*_*NPQr*_), and (o) rate constant of total non‐photochemical quenching (*K*_*NPQ*_). All parameters are after Porcar‐Castell (2011)—equations available in appendices (Equations (A1), (A2), (A3), (A4), (A5), (A6), (A7), (A8), (A9), (A10), (A11)). Wilcoxon rank‐sum *p*‐value: *p < 0.1, **p < 0.05, ***p < 0.01, ns = nonsignificant.

The sustained PQ parameter qLs separated significantly (Wilcoxon rank‐sum *p* < 0.05) by group at the end of WS and remained so during recovery (Figure A2g), while the reversible PQ component qLr separated only during the height of drought (Figure A2h). Like qLs, total PQ parameter qLT was significantly different during the height of WS and post‐WS (Figure A2i). The quantum yield of sustained NPQ (Φ*NPQ*_*s*_) between WS and control plants did not separate during treatment. Φ*NPQ*_*s*_ remained stable for both groups and ranged between near zero and 0.16 (Figure A2j). The difference between WS and control Φ*NPQ*_*R*_ and Φ*NPQ* was nonsignificant on pre‐WS and recovery, but significant on height of WS (Figure A2k,l). NPQ rate constants followed the same trend at a 90% confidence level (Figure A2m–o).

### 3.3 Photosynthetic Gas Exchange and Pigments

**Figure A3:**
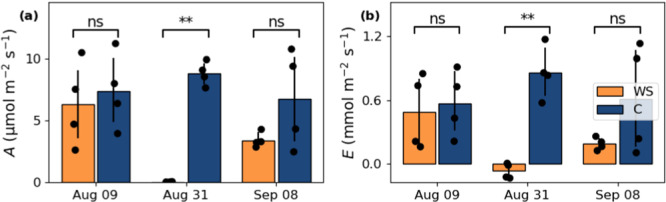
Gas exchange variables (a) CO_2_ assimilation rate *A* (µmol m^−2^ s^−1^), and (b) transpiration rate *E* (mmol m^−2^ s^−1^) on 3 days representing pre‐WS (August 9), height of WS (August 31) and recovery (September 8). Wilcoxon rank‐sum *p*‐value: *p < 0.1, **p < 0.05, ***p < 0.01, ns = nonsignificant.

The CO_2_ assimilation rate *A* was similar between control and WS during pre‐WS (August 9). WS plant *A* was near‐zero at the end of WS (August 31) and recovered to the low‐end‐range of pre‐WS values by the end of recovery (September 8) (Figure A3a). Transpiration rate *E* was similarly near‐zero for WS plants during the height of WS and increased slightly by the end of the recovery period (Figure A3b).

**Figure A4:**
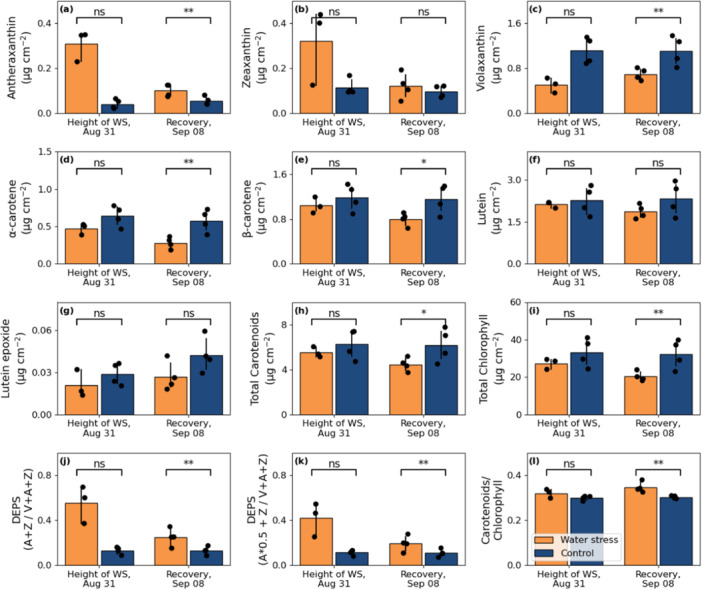
Concentrations of pigments (a) antheraxanthin (A), (b) zeaxanthin (Z), (c) violaxanthin (V) and the de‐epoxidation state of the xanthophyll cycle (DEPS) as (d) A + Z/V + A + Z and (e) alternatively as A*0.5 + Z/V + A + Z after Demmig‐Adams and Adams (1996). Concentration of (f) α‐carotene, (g) β‐carotene, (h) lutein (L), (i) lutein epoxide (Lx), (j) all available carotenoids (A, Z, V, L, Lx, neoxanthin, α‐carotene, and β‐carotene), (k) chlorophyll a and b, and (l) ratio of all carotenoids to chlorophylls. Concentrations are given as µm cm^−2^, and ratios as mol per sample/mol per sample. Wilcoxon rank‐sum *p*‐value: *p < 0.1, **p < 0.05, ***p < 0.01, ns = nonsignificant.

In comparison to controls, the DEPS was higher in WS plants on the first sampling point at the height of WS (the reason that pigment differences were not statistically significant was probably due to the small sample sizes, Figure A4). Consequently, DEPS decreased for all WS plants and stayed stable for controls from the WS period to the second sampling day at the end of the recovery period (Figure A4d,e). Nevertheless, in terms of DEPS WS plants did not fully recover to the level of control plants. By the end of the study, the concentrations of antheraxanthin (A) (Figure A4a) and zeaxanthin (Z) (Figure A4b) decreased for WS plants whereas the concentration of violaxanthin (V) increased for WS but remained stable for control plants (Figure A4c).

Total chlorophyll concentration was lower for WS plants on the first sampling point, reducing even further after the resumption of watering. Controls remained approximately constant across the days (Figure A4g). Hence the total concentration of both carotenoids (Figure A3j) and chlorophylls (Figure A4k) decreased for WS plants from height of WS to the second sampling point which was at the end of recovery. The carotenoid/chlorophyll ratio increased indicating that the relative loss of chlorophylls outweighed the loss of carotenoids (Figure A4l).

### 3.4 Imaging Spectroscopy

**Figure 3:**
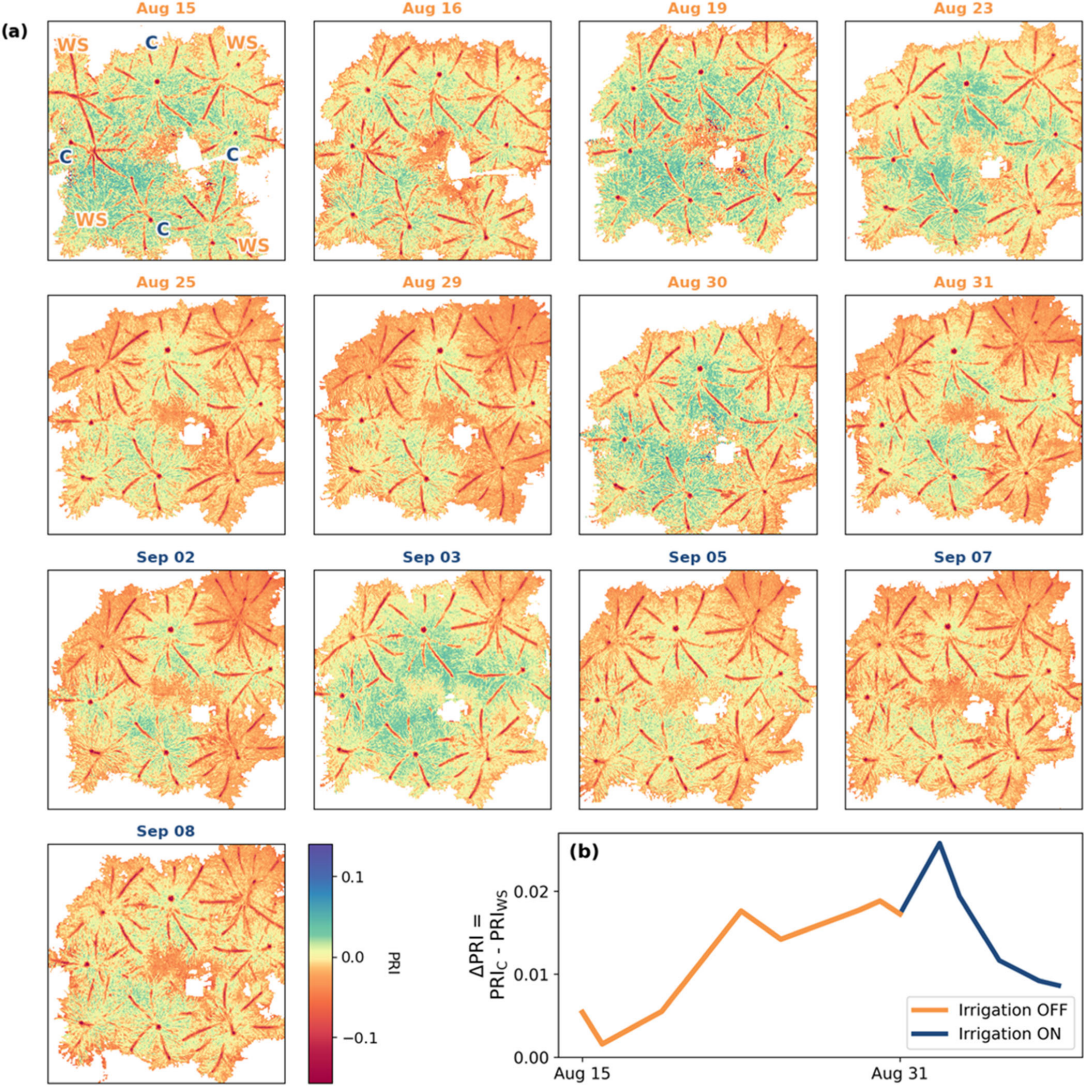
(a) PRI images and (b) a timeseries of the development of PRI difference (ΔPRI = PRI_C_ – PRI_WS_) between WS and control plant average based on primary shoot circle masks. NDVI‐based filter (NDVI < 0.25) was applied to images for visual clarity. Labels in image (a) August 08 are ‘C’ for control and ‘WS’ for water stress. [Color figure can be viewed at wileyonlinelibrary.com]

**Figure A5:**
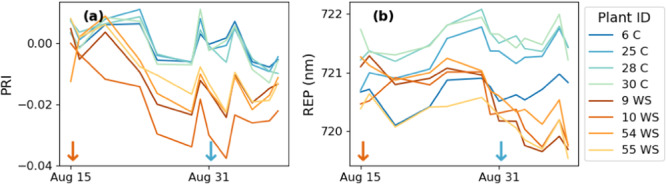
Timeseries of average (a) PRI and (b) REP by plant averaged over a circle mask (Figure 1d) containing the primary shoot.

The distribution of mean PRI values diverged significantly (determined by Wilcoxon sum‐rank test, *p* < 0.05) between WS and control plants starting from the 23rd of August, which was the 8th day of water limitation treatment, until the conclusion of the experiment (Figure 3b). Due to the day‐to‐day fluctuation of temperature, light environment, and timing of measurements, the variance of PRI values between different days was occasionally large (Figure A5a). Nonetheless, the separation between WS and control PRI within the WS days remained discernible after 23rd of August, as the temporal noise pattern was similar in all plants. The PRI difference was most pronounced at the end of the WS period and at the start of recovery monitoring period as observed on the 31st of August and 2nd of September (Figure 3b). With a 2‐day delay after irrigation was restarted, PRI recovered to some extent from the 3rd of September on (Figure 3a,b). Differences between individual control plants in terms of PRI during the experiment were small, whereas WS plants showed a larger difference between individual trees (Figure A5a).

**Figure 4:**
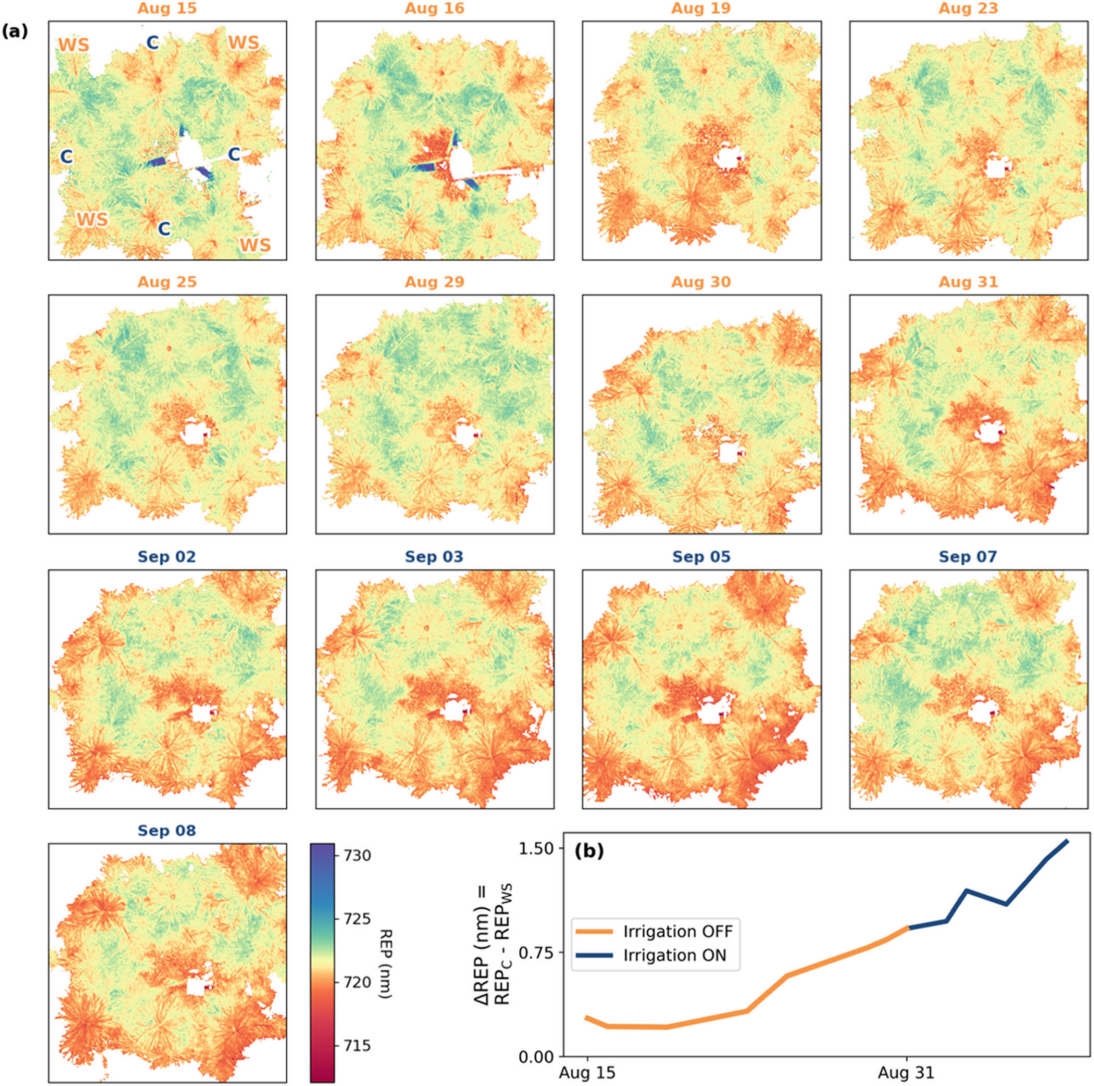
(a) REP images and (b) a timeseries of the development of REP difference (ΔREP = REP_C_ – REP_WS_) between WS and control plant average based on primary shoot circle masks. NDVI‐based filter (NDVI < 0.25) was applied to images for visual clarity. Labels in image (a) Aug 08 are ‘C’ for control and ‘WS’ for water stress. [Color figure can be viewed at wileyonlinelibrary.com]

The REP values of WS plants ranged between 719.6 and 721.4 nm, while control plant REP ranged from 720.1 to 722.1 nm. Control and WS plants separated significantly (according to Wilcoxon sum‐rank test, *p* < 0.05) on the 2nd of September, and remained so till the end of the experiment, although with the exception of one control plant, plant 6 C, control plants separated from WS plants already at mid‐WS treatment (Figure A5b). The continuing divergence of control and WS plants during recovery monitoring was evident in the REP of all control plants, including plant 6C, remaining stable as WS REP continued to decline. The difference in mean REP between WS and control plants (ΔREP) was stable at the beginning of the experiment but increased from mid‐WS till the end of the experiment (Figure 4b).

**Figure 5:**
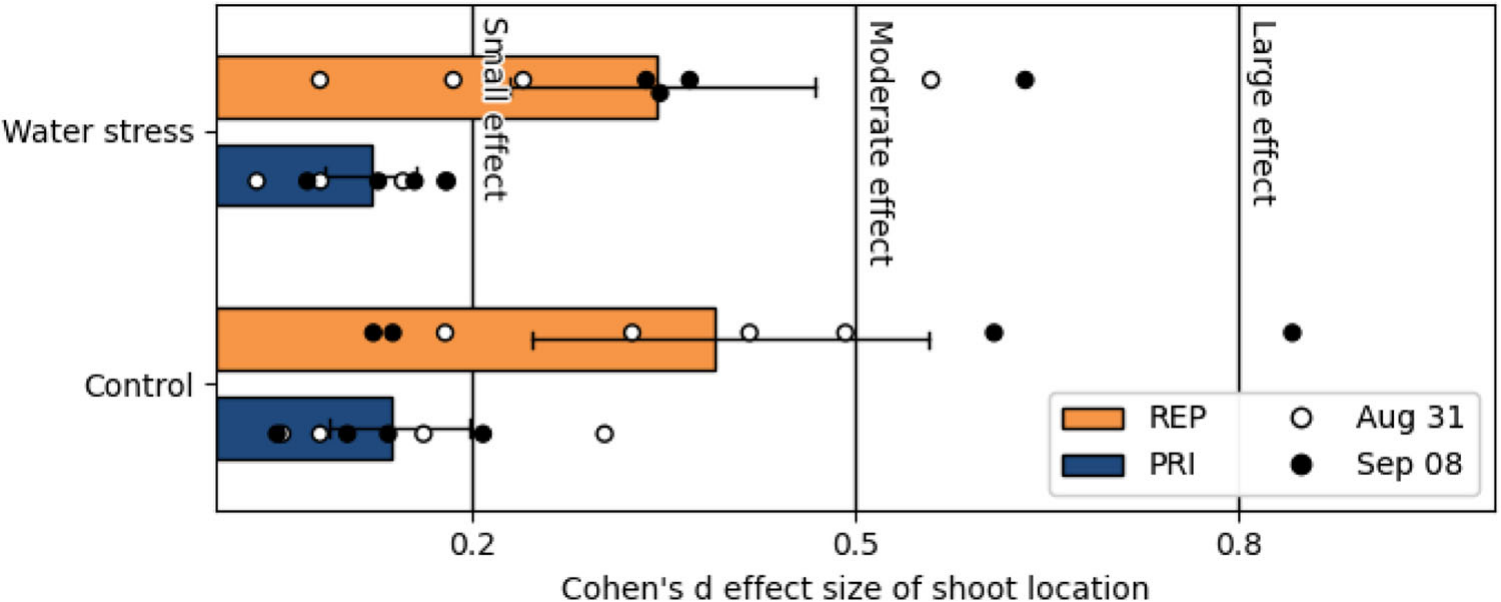
Cohen's *d* effect size of shoot location (primary vs. lateral) on optical indices PRI and REP on August 31 and September 8. [Color figure can be viewed at wileyonlinelibrary.com]

The effect size of shoot position, that is, whether the shoot was the primary or a lateral shoot, on the value of REP was larger than on PRI. On REP, Cohen's *d* effect size varied between small and large, whereas on PRI it was consistently small regardless of water stress (Figure 5). Overall, REP values of needles pointing away from neighbouring saplings had lower REP in comparison to inner and in‐between lateral shoots. Visually this is seen as a ring‐effect that is especially strong in all WS plants from the 31st of August till the end of the experiment (Figure 4a, images August 31 to September 08).

### 3.5 Relationships Between HSI and Other Parameters

**Figure 6:**
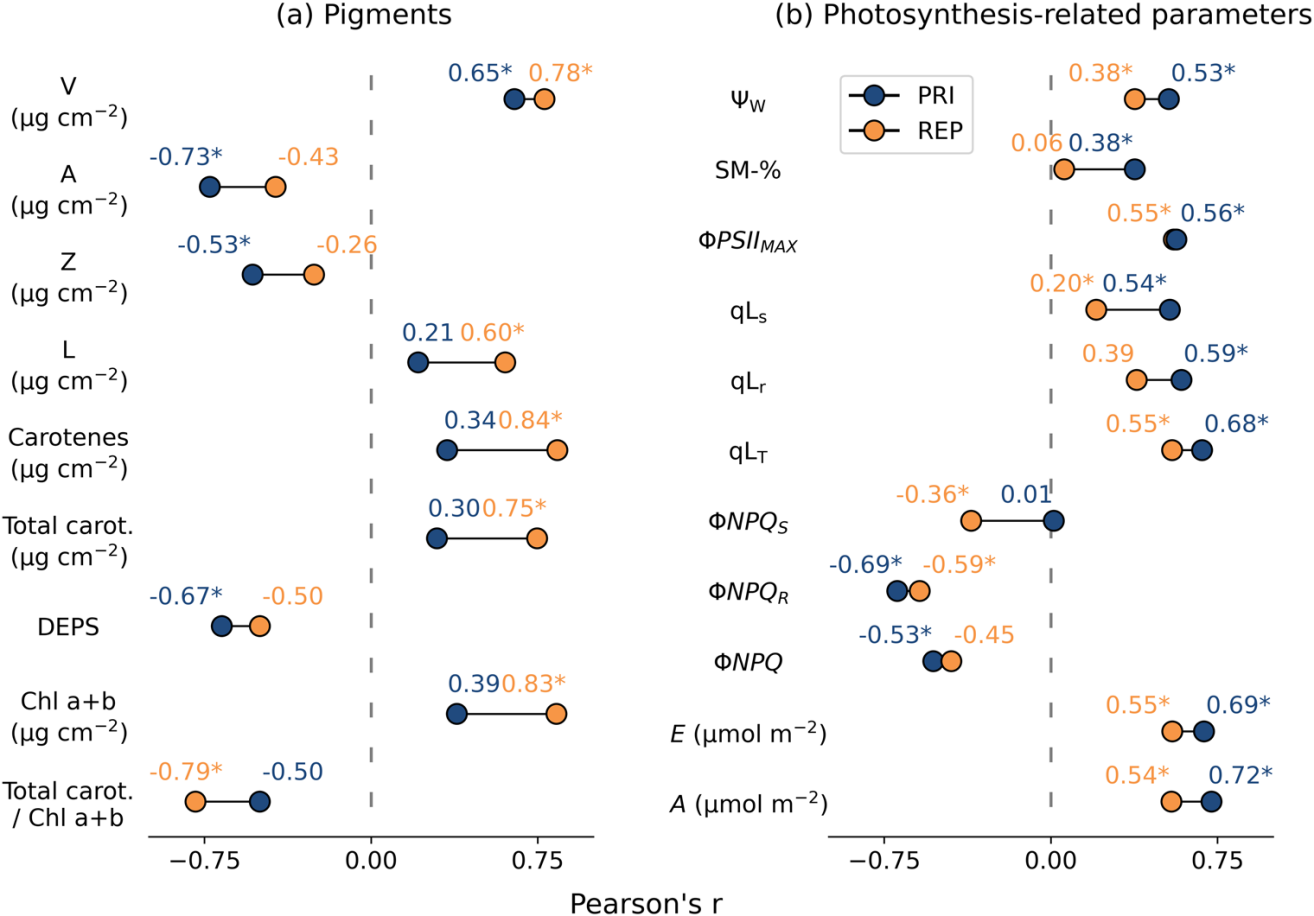
Pearson's *r* correlation coefficient of spectral indices PRI and REP with (a) pigments violaxanthin (V), antheraxanthin (A), zeaxanthin (Z), lutein (L), α‐ and β‐carotene (carotenes), total carotenoids (V, A, Z, L, Lutein epoxide, neoxanthin, α‐ and β‐carotene), DEPS (A + Z/V + A + Z), chlorophyll a and b, and with (b) photosynthesis‐related and other parameters, including leaf water potential (*Ψ_W_*), relative soil moisture (SM‐%), maximum quantum yield of PSII photochemistry (Φ*PSII*_*MAX*_), sustained photochemical quenching parameter qLs, reversible photochemical quenching parameter qLr, total photochemical quenching parameter qLT, yield of sustained non‐photochemical quenching (Φ*NPQ*_*S*_), yield of reversible non‐photochemical quenching (Φ*NPQ*_*R*_), yield of total non‐photochemical quenching (Φ*NPQ*), transpiration rate (*E*), and CO_2_ assimilation rate (*A*). PRI and REP were based on average value over a circle mask. *p < 0.05. [Color figure can be viewed at wileyonlinelibrary.com]

PRI had a significant (*p* < 0.05) relationship with all xanthophyll pigments. The relationship was negative with the de‐epoxified antheraxanthin (*r* = −0.73) and zeaxanthin (*r* = −0.53), and positive with violaxanthin (*r* = 0.65). Consequently, DEPS (Equation 1) had a moderate negative (*r* = −0.67) relationship with PRI (Figure 6a). PRI did not correlate significantly with other pigments. REP had a strong and significant relationship with the total concentration of chlorophyll (*r* = 0.83), and chlorophyll *a* (*r* = 0.84) and *b* (*r* = 0.82) individually. REP also correlated with some carotenoids, notably neoxanthin (*r* = 0.75), violaxanthin (*r* = 0.78) and carotenes (*r* = 0.84) (Figure 6a).

**Figure A6:**
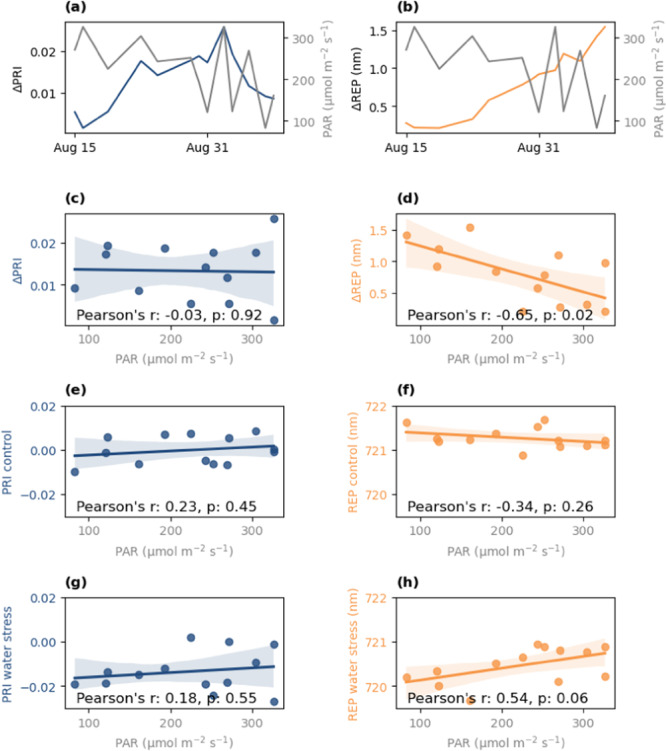
Timeseries of PAR in the greenhouse at the time of HSI with difference between average control and water stressed (a) PRI (ΔPRI) and (b) REP (ΔREP). Linear regression of PAR in the greenhouse at the time of HSI with (c) ΔPRI, (d) ΔREP, (e) average control PRI, (f) average control REP, (g) average water stressed PRI, and (h) average water stressed REP.

Leaf water potential *Ψ_W_* correlated with both PRI and REP moderately (*r* = 0.53 and *r* = 0.38, respectively). PRI correlated moderately with PQ parameters qLs (*r* = 0.54), qLr (*r* = 0.59) and qLT (*r* = 0.68), and NPQ yield parameter Φ*NPQ_R_* (*r* = −0.69), but not with Φ*NPQ_S_*. REP had a significant (*p* < 0.05) relationship with Φ*PSII*_*MAX*_ (*r* = 0.56), Φ*NPQ_R_* and Φ*NPQ_S_*, albeit the correlation with Φ*NPQ_S_* was weaker (*r* = −0.36). Transpiration rate *E* and CO_2_ assimilation rate *A* had significant relationships with both PRI and REP, but the relationship was stronger with PRI (Figure 6b). PRI was uncorrelated with instantaneous PAR (Figure A6). There was a moderate correlation between PAR and REP in control trees, which was probably spurious due to the declining PAR trend that coincided with the experimental period.

## 4 Discussion

We hypothesized that subjecting *P. sylvestris* to sustained soil water deficit would result in changes in carotenoid and chlorophyll pigments that were optically measurable in vivo. The imaging spectroscopy results supported this hypothesis. The carotenoid‐related PRI region and the chlorophyll‐induced red edge reflectance dynamics were temporally and spatially distinct over the course of the experiment. Significant WS‐induced changes in the PRI region were detected earlier and partially recovered (Figure 3), whereas red‐edge changes in reflectance continued to heighten even after rewatering (Figure 4). These results support our hypothesis that PRI is able to detect reversible changes related to NPQ whereas REP responded to degradation of chlorophyll.

### 4.1 Water Stress Physiology

*P. sylvestris* is an isohydric species that promptly closes its stomata when soil moisture decreases (Irvine et al. 1998; Poyatos et al. 2008). This restricts loss of water due to transpiration, making *Ψ_W_* more resistant to change (Poyatos et al. 2008). Nonetheless, we observed a substantial drop in WS plant *Ψ_W_* that affirmed extreme water stress. The strict closing of stomata was also evident, as transpiration rate *E* and CO_2_ assimilation rate *A* of WS plants reached near‐zero values at the height of WS. As relative soil moisture increased during recovery, *E* and *A* rates of all WS plants improved, but not to the extent of control plants.

In WS plants, and occasionally control plants, *Ψ_W_* was below −1.5 MPa, beyond which xylem embolism has been reported to increase substantially (Salmon et al. 2015). It is therefore probable that, the WS plants would not be able to fully recover to the level of their pre‐WS hydraulic functioning even if we extended the recovery monitoring period. Indeed, the legacy effect of WS on gymnosperm trees like *P. sylvestris*, which relates to their poor ability to refill embolized xylem tracheids, often persists for years aften an extreme WS event limiting function notably (DeSoto et al. 2020; Rehschuh et al. 2020).

Φ*PSII*_*MAX*_ of WS plants decreased significantly during treatment, and after a 2‐day delay rapidly increased when irrigation was resumed (Figure 2c). The decrease in WS plant Φ*PSII*_*MAX*_ was accompanied by an increase in *F*_0_, while no change in *F*_*M*_ in relation to control plants was detected. This indicates that the change in WS Φ*PSII*_*MAX*_ was due to decreased PSII activity or photoinhibition, rather than increased sustained NPQ. In tandem with these results, we also detected an increase in the yield of reversible NPQ (Φ*NPQ*_*R*_) in WS plants during the peak of WS. Previous *P. sylvestris* studies with various experimental set‐ups have reported a WS‐induced NPQ response (Garcia‐Forner et al. 2016; Zlobin, Ivanov, et al. 2019, Zlobin and Kartashov 2019; D'Odorico et al. 2021), although none have to our knowledge described its temporal components (sustained and reversible) in isolation. Together our results point to a combination of reversible NPQ and photoinhibition as the main impacts of the treatment. It should be noted that the *F*_*M*_, *F*_0_, *F*_*S*_ and *F'*_*M*_ in this study were dependent on leaf area, and accounting for needle gap fraction would improve their estimation and possibly our interpretation (Rajewicz et al. 2019). As a final point, photorespiration can also potentially reduce the sensitivity of these parameters to WS (Flexas, Escalona, and Medrano 1999; Marrs et al. 2020), decoupling PRI from photosynthetic radiation use efficiency (Mulero et al. 2023).

In terms of pigments, the WS treatment caused degradation in the total pool of chlorophyll, and to a lesser extent total carotenoids, which did not recover on re‐watering and was consistent with previous studies (Zlobin, Kartashov, et al. 2019). This may explain the relatively weak correlation between carotenoid/chlorophyll and PRI (Figure 6a) i.e., the slow PRI component (D'Odorico et al. 2021). Elevated values of the de‐epoxidation state of the xanthophyll cycle (DEPS) in WS trees were in line with the hypothesized PRI response. As an aside, PAR in the greenhouse was relatively low compared to outdoor conditions—around 100 μmol m^−2^ s^−1^ on cloudy days to 300 μmol m^−2^ s^−1^ on sunny days. However, the xanthophyll cycle may be de‐epoxidized under stress in low light conditions and in extreme cases, in the dark (Fernández‐Marín et al. 2021). The violaxanthin de‐epoxidase enzyme, which catalyses the de‐epoxidation of violaxanthin to zeaxanthin, is dependent on thylakoid lumen acidification rather than absolute light intensity levels (Jahns, Latowski, and Strzalka 2009). Thylakoid lumen acidification is in turn dependent on the acclimation state of photosynthetic apparatus, especially protein contents of Cyt‐b6f complex and ATP synthase, which are also known to decrease under water stress conditions (Schöttler and Tóth 2014). Recent studies also suggest inhibition of zeaxanthin epoxidase activity during stress‐conditions connected to PSII photoinhibition, in which zeaxanthin epoxidase is degraded alongside photodamaged D1 protein of the PSII reaction centre (Schwarz et al. 2015, Bethmann et al. 2019, Holzmann, Bethmann, and Jahns 2022, Bethmann et al. 2023, Küster et al. 2023). Considering that water‐stress in this study led to significant changes in qLs (Figure A2g) indicative of PSII photoinhibition (Porcar‐Castell 2011), de‐epoxidized xanthophylls might represent a long‐term protective mechanism of light‐stress memory (Bethmann et al. 2019), which is in line with the partial recovery of DEPS, and PRI, on resumption of watering (Figure A4d).

### 4.2 HSI Spectroscopy of Water Stress‐Induced Changes in Photosynthetic Pigments

As hypothesized, we found that PRI was inversely related to DEPS. The correlation between PRI and DEPS in this study was similar to previous canopy level *P. sylvestris* studies (Hernández‐Clemente et al. 2011; D'Odorico et al. 2021). Much like PRI, HSI‐based REP declined during WS, but unlike PRI, it did not recover when irrigation was resumed.

As a result of the temporally distinct PRI and REP patterns, two separate developmental groupings in traits emerged. First, traits that recovered and correlated with PRI, and second, traits that did not recover and correlated with REP. PRI correlated with xanthophylls, and physiological and photosynthetic traits (Figure 6a,b), whereas REP correlated with chlorophylls and some carotenoids (Figure 6a). Although chlorophyll‐related optical indices have been used as a proxy for photosynthetic production (Gitelson et al. 2006), in this case, PRI functioned as a better indicator of photosynthesis as demonstrated by its stronger relationship with CO_2_ assimilation and maximal and light‐adapted PSII quantum yields (Figure 6b).

PRI images of WS plants (Figure 3) revealed a spatial gradient of lower PRI values from top‐right to higher PRI values in the lower left. In control plants, a comparable gradient did not appear. The WS plants with the lowest PRI values were closest to the southerly window that received the most sunlight at the time of imaging (approximately midday), hence the PRI differential or gradient between WS plants was contributed to by the slightly disproportionate amount of light received by each plant. The corner positioning of the WS trees could also have influenced the light environment but as most measurement days included some direct sunlight, albeit transmitted through glass and partially diffused through a semitransparent curtain, it is probable that window proximity was the more important factor.

Spatially, REP has a within‐plant gradient from edge‐ and top‐most needles to inner and lower needles, which takes shape as an outer ring of low values in the REP images (Figure 4). This ring effect exists in WS plants and to a lesser extent in control plants. Although multiple scattering and illumination effects—detailed in the next section—contribute to between‐shoot differences in optical vegetation indices (Mottus and Rautiainen 2013), it is likely that this difference is an indicator of differences in chlorophyll concentration within the plant due to the strong relationship between red edge indices and chlorophyll established here and in previous literature (Horler, Dockray, and Barber 1983; Filella and Peñuelas 1994; Frampton et al. 2013). Where PRI responds to current, and immediately preceding, illumination conditions as is evident from the drastic difference in absolute PRI between days and well‐established diurnal cycle (Gamon, Peñuelas, and Field 1990, 1992), we speculate that REP responded to long‐term light conditions as the tallest and outermost shoots were provided the least shade by neighbouring saplings.

### 4.3 Issues Relating to Greenhouse‐Based Imaging Spectroscopy

Reflectance spectroscopy requires the estimation of incident irradiance, which usually varies between measurements when solar radiation is used as the source. We used a horizontally levelled near‐Lambertian reference panel to estimate irradiance (Figure 1a). This method assumed that irradiance at the surface of interest is equivalent to the irradiance arriving at the panel. This assumption is violated for trees which have complex 3D geometries. Further, a large portion of measured radiation is not singly scattered back to the sensor but subject to multiple interactions with canopy and neighbouring elements, for example, greenhouse infrastructure, and the white panel itself may be contaminated by multiple scattering when the panel is set close to the trees as was the case in our study. These multiple scattering interactions are wavelength dependent (Ihalainen and Mõttus 2022). Additionally, some of the measured reflectance also originates from specular effects which do not contain information relating to plant pigments.

Relative to the simple panel correction method here, more sophisticated methods have been applied to close range HSI data to account and correct for these geometrical and structural‐optical effects. For example, some studies have corrected HSI data with 3D measurements of plant geometry and leaf inclination angles which largely control single scattering and shadows (Behmann et al. 2016; Vigneau et al. 2011). Mohd Asaari et al. (2018) developed an empirical correction to account for changes in illumination, multiple scattering, and specular reflectance in water‐stressed maize without the need for additional structural information. Ihalainen, Juola and Mõttus (2023) developed a single‐image correction based on radiative transfer theory which was subsequently applied to outdoor imagery of a shrub.

What all these methods have in common is that they have been applied to broadleaf species. To apply such a method to *P. sylvestris* requires consideration of the intermediate shoot scale (and not just foliar and tree geometry) and the multiple scattering effects due to shoot structure, which is beyond the scope of this study. As to the implications of these effects in the current study, some of the spatial variability observed in Figures 3 and 4 could be related to illumination effects rather than variance in foliar pigment content. It is also possible that a structural/illumination‐related WS effect influenced the observed differences in optical indices. For example, an increased proportion of woody area could modulate PRI as could turgor‐related changes in branch and shoot angles (Junttila et al. 2022). Without detailed structural measurements, these effects are difficult to quantify, hence future studies should include structural observations if possible.

A final limitation relates to the spectral resolution of the HSI instrument used in this study. The shift in absorbance related to xanthophylls extends from approximately 500–570 nm, with a main feature at 531 nm, with additional secondary features within the aforementioned range (Gamon, Serrano, and Surfus 1997, Van Wittenberghe et al. 2019). In this study, the full‐width at half maximum of the PRI bands centred at 531 and 570 nm were 13 and 12 nm, respectively. This means that although we did see differences between control and WS plants that were correlated with the xanthophyll cycle state, it is not possible to disentangle the multiple features that contribute to the broad changes measured here. Future study would benefit from a higher‐resolution camera.

### 4.4 Transferability to Remote Sensing

Despite the limitations above, our results demonstrate that the impacts of WS are detectable in greenhouses with the proviso that appropriate spectral features are selected. In the introduction, we also suggested that our results would be of importance to remote sensing. First, the results add to the evidence that the PRI can be used to detect WS in Scots pine (D'Odorico et al. 2021). Second, the results show that red edge or chlorophyll‐based indices can detect WS with the proviso that such changes may reflect nonreversable physiological changes or permanent damage. The differential response of chlorophyll and xanthophyll cycle pigments during WS is a topic that could benefit from further investigation in Scots pine and other important species.

The recent miniaturization and proliferation of hyperspectral and multispectral imaging technologies coupled with the rise of drones has led to the emergence of the remote sensing of plant individuals (Kellner et al. 2019). Remote sensing at these scales opens new avenues of research, perhaps the most obvious example being high throughput phenotyping. Individual‐scale HSI data can also be used to probe fundamental ecophysiological questions. In this study, we explored whether there was any variation of PRI and REP within individuals. Many traits such as chlorophyll vary with light environment (Niinemets et al. 1998) and leaf age (Wang et al. 2020). The response of PRI to changing light environment has also been previously noted with greater pigments pools and relative response in more light exposed positions (Gamon and Berry 2012).

We did not find spatial responses in PRI within individuals. This may be because, we only analysed primary and secondary shoots from same‐year needles and young trees due to the near‐nadir view angles. It could also be because the PRI data was relatively noisy due to the limitations of the imaging device and our measurement protocols which obscured any minor changes that may have been present. On the other hand, there were positional differences found in the REP (Figure 5). We speculated that these differences probably related to the longer‐term light exposure/environment as those shoots with shorter REPs, or less chlorophyll, were part of the external ring structure evident in Figure 4.

**Figure A7:**
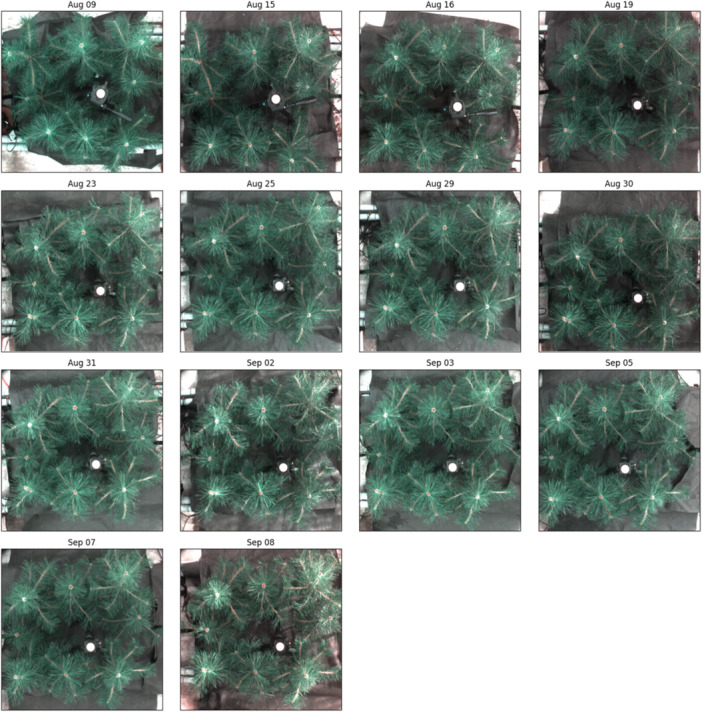
RGB images constructed from HSI. Band centres for red, green and blue were 628.6, 531 and 514.3 nm respectively.

A final noteworthy observation on transferability related to the visibility of branches in the PRI images (Figure 3a), as they are not so evidently visible in the REP (Figure 4a) and RGB images (Figure A7). If branches are not excluded by masking, the PRI will be skewed more negatively than is representative of leaf‐level reality. This poses implications on the reliability of absolute PRI values derived from lower‐resolution imaging spectroscopy typical of moving platforms.

## 5 Conclusions

Our results support the hypothesis that strong WS impacts both chlorophyll and carotenoid‐related optical properties in *P. sylvestris*. As hypothesized, both the PRI and REP responded to WS which went together with photosynthetic downregulation. On re‐watering, the partial recovery in PRI, which did not occur in REP, suggested that although either index may be transferable to WS or drought remote sensing and greenhouse studies, there is value in measuring across the spectrum. We also found wavelength‐dependent spatial patterns in optical properties. These responses were probably related incident irradiance—contemporaneous, in the case of PRI, and longer‐term, in the case of REP. Future work would benefit from a more sophisticated analysis to disentangle the physical causes—3D structure and illumination issues—from the physiological implications of such patterns.

## Conflicts of Interest

The authors declare no conflicts of interest.

## Data Availability

The data that support the findings of this study are openly available in Data from: Hyperspectral imaging reveals differential carotenoid and chlorophyll temporal dynamics and spatial patterns in Scots pine under water stress at https://zenodo.org/records/13985469, reference number 10.5281/zenodo.13985469. Data and software used in the study is available and hosted on Zenodo using DOI (10.5281/zenodo.13985469).

## 1

(A1)
$${\Phi \mathrm{PSII}}_{\mathrm{MAX}}=\frac{F_{M}-F_{0}}{F_{M}},$$

(A2)
$$\Phi \mathrm{PSII}=\frac{F_{S}^{\prime }-F_{M}^{\prime }}{F_{M}^{\prime }},$$

(A3)
$$\Phi {\mathrm{NPQ}}_{S}=\frac{F_{0}}{F_{M}}-\frac{F_{0}}{F_{\mathrm{MR}}},$$

(A4)
$$\Phi {\mathrm{NPQ}}_{R}=\frac{F_{S}}{F_{M}^{\prime }}-\frac{F_{0}}{F_{M}},$$

(A5)
$$\Phi \mathrm{NPQ}=\frac{F_{S}}{F_{M}^{\prime }}-\frac{F_{S}}{{F\prime }_{\mathrm{MR}}},$$

(A6)
$${\mathrm{qL}}_{S}=\frac{\frac{1}{F_{0}}-\frac{1}{F_{M}}}{\frac{1}{F_{0R}}-\frac{1}{F_{\mathrm{MR}}}},$$

(A7)
$${\mathrm{qL}}_{R}=\frac{\frac{1}{F_{S}}-\frac{1}{F_{M}^{\prime }}}{\frac{1}{F_{0}}-\frac{1}{F_{M}}},$$

(A8)
$${\mathrm{qL}}_{T}=\frac{\frac{1}{F_{S}}-\frac{1}{F_{M}^{\prime }}}{\frac{1}{{F\prime }_{0R}}-\frac{1}{{F\prime }_{\mathrm{MR}}}},$$

(A9)
$$K_{\mathrm{NPQs}}=\frac{F_{\mathrm{MR}}}{F_{M}}-1,$$

(A10)
$$K_{\mathrm{NPQr}}=\frac{{F\prime }_{\mathrm{MR}}}{F_{M}^{\prime }}-\frac{F_{\mathrm{MR}}}{F_{M}},$$

(A11)
$$K_{\mathrm{NPQ}}=\frac{{F\prime }_{\mathrm{MR}}}{F_{M}^{\prime }}-1,$$

(A12.)
$$F_{0R}=(1-\Phi {\mathrm{PSII}}_{\mathrm{ref}})\times F_{\mathrm{MR}}.$$
